# Organizing the Confusion Surrounding Workaholism: New Structure, Measure, and Validation

**DOI:** 10.3389/fpsyg.2017.01803

**Published:** 2017-10-19

**Authors:** Or Shkoler, Edna Rabenu, Cristinel Vasiliu, Gil Sharoni, Aharon Tziner

**Affiliations:** ^1^School of Behavioral Sciences, Netanya Academic College, Netanya, Israel; ^2^Department of Business Consumer Science and Quality Management, The Bucharest University of Economic Studies, Bucharest, Romania

**Keywords:** workaholism, facet, work drive, measurement, research methods, cultural differences

## Abstract

Since “workaholism” was coined, a considerable body of research was conducted to shed light on its essence. After at least 40 years of studying this important phenomenon, a large variety of definitions, conceptualizations, and measures emerged. In order to try and bring more integration and consensus to this construct, the current research was conducted in two phases. We aimed to formulate a theoretical definitional framework for workaholism, capitalizing upon the Facet Theory Approach. Two basic facets were hypothesized: A. Modalities of workaholism, with three elements: cognitive, emotional, and instrumental; and B. Resources of workaholism with two elements: time and effort. Based on this definitional framework, a structured questionnaire was conceived. In the first phase, the new measure was validated with an Israeli sample comparing two statistical procedures; Factor Analysis (FA) and Smallest Space Analysis (SSA). In the second phase, we aimed to replicate the findings, and to contrast the newly-devised questionnaire with other extant workaholism measures, with a Romanian sample. Theoretical implications and future research suggestions are discussed.

## Introduction

Since the early 1970s there have been concrete and strong testimonies to the centrality of work in people's lives (Arvey et al., 2004), much beyond being only an economical consideration (Highhouse et al., 2010). As evidence, the majority of people would still continue working regardless of their economic status (NRC, 1999). The experience of working is vastly more important than the job itself, and this also explains why many of us devote most of our waking hours to work, beyond any other human activity (for further reading, see Landy and Conte, 2016).

In recent years there has been a considerable increase in the time invested in work, also as a byproduct of the greater accessibility to technology and industrial competition (Lee et al., 2007). Regardless of this trend, research has found individual differences in the devotion of time to work. One of the pioneering works that tried to address those differences was Oates' (1971) research on workaholism.

Oates (1971) coined the term “workaholism” and defined the phenomenon as “… an addiction to work, the compulsion or uncontrollable need to work incessantly” (Oates, 1971, p. 11). Oates noted that workaholics' need to work becomes exaggerated and may cause harm to their health, personal happiness, interpersonal relations, and social functioning. In a later discussion of the term, Spence and Robbins (1992) regarded workaholism as an addiction. They noted that “the workaholic feels driven or compelled to work, not because of external demands or pleasure in work, but because of inner pressures that make the person distressed or guilty about not working” (p. 161). Since Spence and Robbins (1992), there have been many papers in the academic literature devoted to workaholism (e.g., Schaufeli et al., 2008; Patel et al., 2012; Andreassen et al., 2014). Most researchers agree upon workaholism's core behavioral manifestation, namely, heavy investment in work (Spence and Robbins, 1992; Scott et al., 1997; Snir and Harpaz, 2015). That is to say, workaholics spend many hours a week on work-related activities when given the opportunity to do so (Snir and Zohar, 2008), and much beyond what is required or expected by colleagues or organizational demands (Scott et al., 1997). However, in a recent meta-analysis (Clark et al., 2016), the authors argued that “there continues to be confusion surrounding the definition, conceptualization, and measurement of workaholism, which has resulted in diverging opinions…” (p. 2).

Consequently, workaholism has been addressed with vague conceptual definitions and operationalizations, lacking compelling theoretical frameworks and sufficient studies in this regard. Moreover, there are several overlapping concepts of workaholism such as passion to work, job engagement, job involvement, and more (Andreassen, 2015; see also McMillan and O'Driscoll, 2006). As Clark et al. (2016) concluded in their recent meta-analysis:

… we also encourage the development of new measures of workaholism derived deductively using the largely agreed-on themes relating to the definition of workaholism, rather than the continued use or modification of existing scales that may not fully assess this multifaceted construct (or that examine additional factors that are not necessarily aspects of workaholism) and/or have consistently fared poorly when subjected to factor analyses and other psychometric analyses (e.g., Spence and Robbins's Workaholism Battery) (p. 31).

In light of operationalization and conceptual difficulties, we applied the Facet Theory. It attempts to formally define the universe of observations and to test hypotheses about the relationship between the definitional framework and the structure of the empirical observations (Elizur, 1984). Facet theory is a method by which the components of a problem or the issue under investigation can be defined formally (Guttman, 1957), and it allows for depicting a complex interplay of variables (Hackett, 2014a). A facet is a group of common traits that represents semantic components of a context field (Yaniv, 2011).

In the present study we attempted to develop a framework of workaholism. Based on the literature, we distinguished two basic facets to define workaholism: A—modalities of workaholism, and B—resources of workaholism.

### Facet A–modalities of workaholism

“Workaholics are those whose emotions, thoughts, and behaviors are strongly dominated by their work” (Ng et al., 2007, p. 114). As mentioned, workaholism requires an investment of cognitive energy. The workaholic is driven to allocate a vast amount of thoughts into his or her work (Snir and Zohar, 2000), being overly concerned with it (Andreassen et al., 2014), or even persistently thinking about work when not working (Scott et al., 1997). Therefore, we defined the first element: a1—cognitive.

One of the dimensions of workaholism suggested by Ng et al. (2007) was the affective one, and, indeed, in most of the existing measures of workaholism, the emotional aspect is very clearly addressed (e.g., Spence and Robbins, 1992; Robinson, 1998; Schaufeli et al., 2009; Andreassen et al., 2012). However, it was not fully theoretically defined until the current study, as far as we know, and even in a recent meta-analysis, Clark et al. (2016) encourage conducting future research on the emotional aspect of workaholism “… as we really do not know enough about the affective nature of workaholism based on the extant literature” (p. 31). It is important to note that the emotions surrounding workaholism can be both positive (e.g., enthusiasm about working) and negative (e.g., frustration about not working). Therefore, we defined the second element: a2—emotional.

Another dimension of workaholism suggested by Ng et al. (2007) was the behavioral one (we prefer to call it—instrumental). The act of working (rather than the nature of the work itself, Ng et al., 2007, p. 114) is fundamental for the definition of workaholism (e.g., Schaufeli et al., 2009), and also explains why workaholics are considerably invested in work activities (Snir and Zohar, 2000). Therefore, we defined the third element: a3—instrumental.

### Facet B–resources of workaholism

There is mild consensus in a body of research that has emphasized long working hours as the critical component of workaholism's definition (e.g., Oates, 1971; Spence and Robbins, 1992; Scott et al., 1997; Ng et al., 2007). Mosier (1983) even defined workaholism as working over 50 h a week. Therefore, we defined the first element: b1—time.

Effort in work is the quantity and quality of physical activities invested in the job (e.g., Gorman and Kmec, 2007). It is clear that while many measures emphasize the time aspect of workaholism, only few, in actuality, regarded the effort invested in work. It is notable that those who referred to effort did so by addressing it as “working hard” (e.g., Spence and Robbins, 1992; Schaufeli et al., 2009). This necessitates a renewed reference to the effort aspect of workaholism, and, indeed, recently, Snir and Harpaz (2012) included this aspect of workaholism in their study. Therefore, we defined the second element: b2—effort.

### Mapping sentence

**Figure 1:**
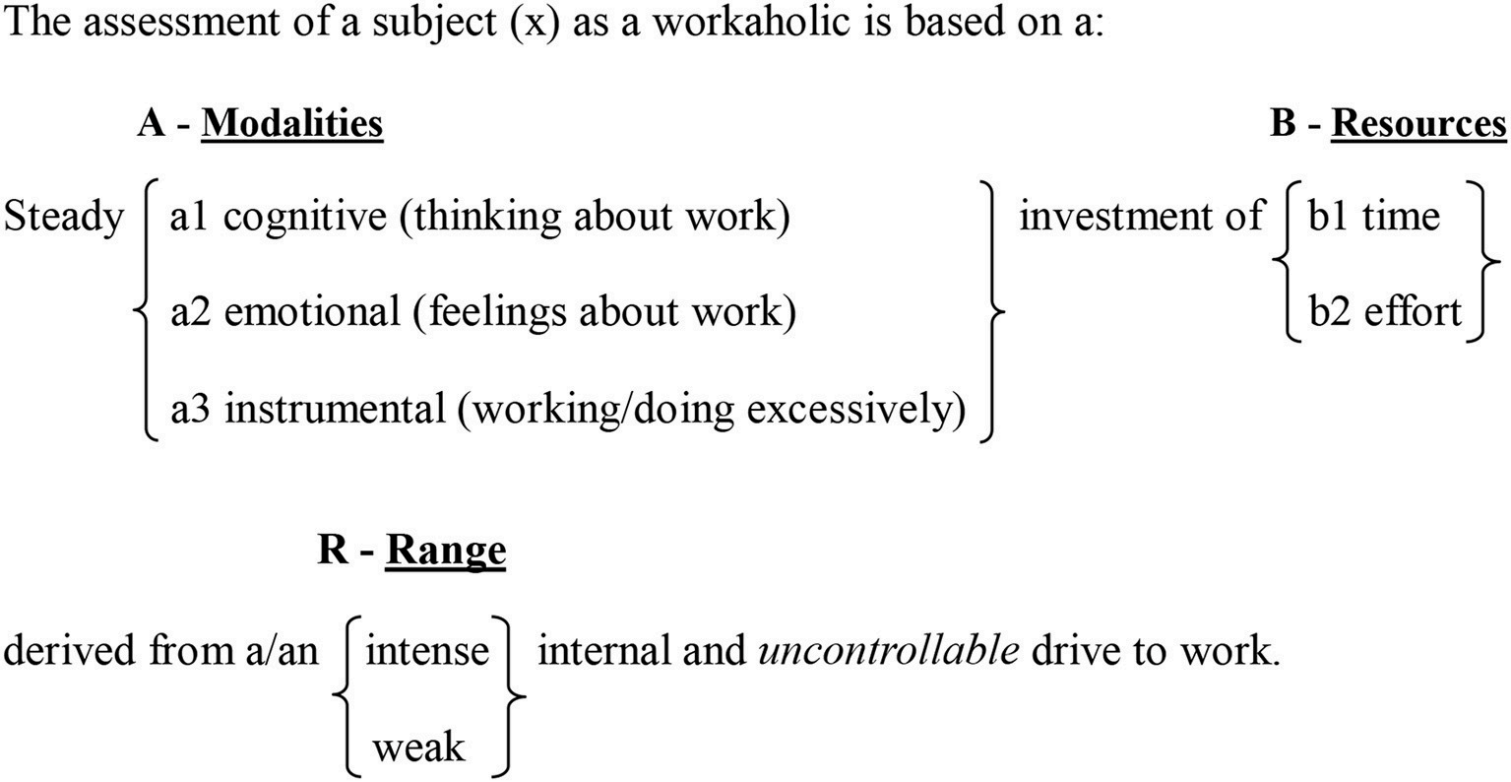
Mapping sentence definition of workaholism.

“A mapping sentence allows formal and exacting consideration of the variables that comprise a research domain” (Hackett, 2014b, p. 67), and is the heart of the facet theory approach (Fisher, 2011; Hackett, 2014b). This sentence serves as a guide for formulating hypotheses, creating structured assumptions, planning and collecting observations, and analyzing data (Levy, 2005; Fisher, 2011). It provides a sound basis for the empirical associations between observed variables (i.e., different facets and elements) (Fisher, 2011). The following mapping sentence (see Figure 1) presents the definitional framework suggested for workaholism. The Cartesian product of the facet elements provided 3 × 2 = 6 combinations, upon which our new measure was based (see Method section). Workaholism will be sampled methodically by creating three items for each combination resulting in 18 items in total. The created items were consequently used to build a facet questionnaire of workaholism (see Appendix B).

Thus, we hypothesize:

**H1—**The empirical results will reflect the components of the concept of workaholism, as defined in the mapping sentence. A distinct area will be found for each facet and each element.

**H2—**The workaholism modalities facet (A) will have a polarizing role, in which its elements are away from the center (the origin) in a different direction in the geometric space (Elizur, 1984). This is a general facet and can be found in many behavioral sciences papers. Depending on the hypotheses and the research context, this facet can fulfill various roles: a modulating role (e.g., Elizur and Tziner, 1985; Sagie, 1995) or a polarizing role (e.g., Elizur, 1984, 1991; Rabenu et al., 2015b). We found no base to organize the elements in this facet in a particular order, and thus we assumed it to be a polarizing facet.

**H3—**The workaholism resources facet (B) will be modulating. A modulating facet organizes the elements from the central area to the peripheral area in the geometric space (Elizur, 1984). The higher the proximity between the items, the closer the region will be to the center of the dimensional map. Even though time and effort can be regarded subjectively, it is the time aspect which is more generally agreed upon due to its universal measurement scale (i.e., seconds, minutes, working hours, formal work break, etc.). Because of this universal understanding of time, we assumed that the time items would be more closely related to each other. Thus, we assumed that the elements in this facet will be ordered so that the time aspect is central and the effort aspect is peripheral.

**H4—**The total structure obtained from facets A and B will be a circular-radial formation. As such it is called *radex*, and is created by the combination of polarizing and modulating facets (Guttman, 1954; Elizur, 1984).

**Figure 2:**
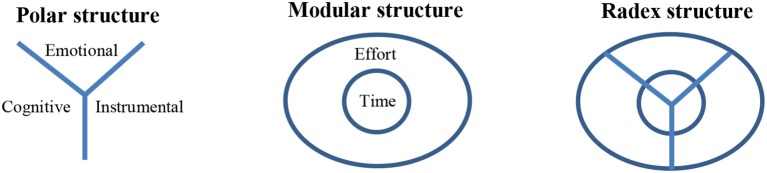
The roles of the facets.

The current paper, as stated, is based on two samples. In the first phase we validate our new measure with a convenience sample from Israel, and then, in the next phase, try to replicate the results in a sample from Romania for the hypothesized facets' structures see figure 2. We have chosen Romania because it is an ex-communist state in Central and Eastern Europe (CEE) that joined the European Union only a decade ago (in 2007). Romania, a country currently in transition from a centrally-planned economy to a free-market economy, offers a unique and interesting focus since “little is known about the possibilities of applying Western conceptual models in an eastern European context” (Buzea, 2014, p. 426). In the communist era, the labor market was heavily regulated and the main objective was full employment, irrespective of whether it was productive or not (Parlevliet and Xenogiani, 2008). The new labor code established in Romania in 2003 introduced important changes with respect to the types of labor contracts that can be recognized (e.g., part-time and fixed-term). At the same time, there is vast informal employment in Romania (Parlevliet and Xenogiani, 2008). We believe that the “virgin soil” in Romania, translated into the actual possibility of making a considerable investment in work (in terms of time and effort), and the probability that addiction to work is manifested, is worth investigating.

## Method

### Participants

We used two samples in our study—166 Israeli workers and 1,117 Romanian MBA working students. The demographic data is presented in Table 1.

**Table 1:** Demographic information for Israeli and Romanian samples.

**Parameter**	**Category**	**Israel**		**Romania**	
		***N***	**%**	***N***	**%**
Gender	Males	58	34.9	448	40.1
	Females	108	65.1	669	59.9
Age	18–25	24	14.5	606	54.3
	25–35	48	28.9	298	26.7
	35–45	58	34.9	89	8.0
	45–55	28	16.9	103	9.2
	55–65	8	4.8	19	1.7
	65+	–	–	2	0.2
Marital status	Single	49	29.5	418	37.4
	Married/coupled	20	12.0	73	6.5
	Married/coupled + children	91	54.8	417	37.3
	Divorced/separated/widowed	–	–	209	18.7
	Divorced/separated/widowed + children	–	–	579	51.8
Education	High-school	9	5.4	300	26.9
	Tertiary	12	7.2	203	18.2
	Student/graduate of B.A	77	46.4	15	1.3
	Student/graduate of M.A. and above	68	41.0	20	1.8
Tenure	0–5	94	56.6	787	70.5
	5–10	34	20.5	159	14.2
	10–15	15	9.0	67	6.0
	15–20	11	6.6	26	2.3
	20–25	7	4.2	26	2.3
	25+	5	3.0	52	4.7
Team work^a^	No	29	17.5	216	19.3
	Yes	137	82.5	901	80.7
Responsibility^b^	No	85	51.2	842	75.4
	Unit/team manager	55	33.1	163	14.6
	Department manager	16	9.6	69	6.2
	Director	10	6.0	43	3.8
Sector	Public sector	26	15.7	253	22.6
	Union/labor federation	6	3.6	18	1.6
	Private sector	120	72.3	823	73.7
	Nonprofit organization	14	8.4	23	2.1
Industry	High-tech	45	27.1	153	13.7
	Low-tech	28	16.9	316	28.3
	Services	93	56.0	648	58.0

a *Working in a team*.

b *Responsibility for other people's work*.

### Measures

- Our new measure, the *Workaholism Facet-Based Scale* (WFBS) was gauged by 18 items on a Likert scale between 1 (“strongly disagree”) and 6 (“strongly agree”). This measure was based on our mapping sentence (as stated above). The measure is divided into six structuples, and for each one we generated three different items. For example: the structuple a1–b1 (cognitive modality + time resource) is presented by “… think about the job all the time”. The structuple a3–b2 (instrumental modality + effort resource) is presented by “… Work very intensely”.

We used different measures to compare to our *Workaholism Facet-Based Scale* (WFBS, see below) for four main reasons: (1) These measures addressed workaholism as an addiction or work drive, by following its classical definition: “an addiction to work, the compulsion or uncontrollable need to work incessantly” (Oates, 1971, p. 11), and (2) in order to avoid overlapping of measures [for example: the Dutch Work Addiction Scale (DUWAS; Schaufeli et al., 2009) already consists of Spence and Robbins' (1992) Drive dimension], we (3) chose the most recent workaholism scales (Schaufeli et al., 2009; Andreassen et al., 2012), and by this we also (4) avoided wearing the participants down with multiple measures of the same nature. In addition, the table in Appendix A presents the similarities and distinctions among the different workaholism measures and our own (WFBS).

Furthermore, we included the Heavy-Work Investment (HWI; Snir and Harpaz, 2012) scale because it has a common ground with the rest of the measures in our survey, because they all regard the time and effort elements, important for defining workaholism.

- *Demographic items*. In addition to demographic items such as: gender, age, tenure, etc., we added four items for job characteristics: (1) “Do you work in a team?” (No/Yes/Other). The two samples did not differ significantly in this regard: χ^2^ (1, *N* = 1,238) = 0.33, *p* = 0.568, ϕ = 0.02). (2) “Are you responsible for other people's work?” (I am not responsible for other people's work/I am a unit/team manager/I am a department manager/I am a director). The two samples differed significantly: χ^2^ (3, *N* = 1,238) = 45.19, *p* = 0.000, *r*_c_ = 0.19, so that the Israeli participants were more responsible for others' work (48.7%) as opposed to the Romanians (24.6%). (3) “To what extent do you have the freedom/autonomy in setting your amount of work hours?” (Likert-type, 1 = “little extent,” 6 = “large extent”; *Israeli* sample: *M* = 3.81, *SD* = 1.44, *R* = 1–6. *Romanian* sample: *M* = 3.31, *SD* = 1.73, *R* = 1–6). The means differ significantly between the two samples: *t*_(1, 281)_ = 3.48, *p* = 0.001, Cohen's *d* = 0.31. (4) “To what extent do you have the flexibility to decide when to work your amount of work hours?” (Likert-type, 1 = “little extent,” 6 = “large extent”; *Israeli* sample: *M* = 3.70, *SD* = 1.53, Range = 1–6. *Romanian* sample: *M* = 3.22, *SD* = 1.75, Range = 1–6). The means differ significantly between the two samples: *t*_(1, 281)_ = 3.50, *p* = 0.000, Cohen's *d* = 0.30.
- *Dutch Work Addiction Scale* (DUWAS; Schaufeli et al., 2009) was gauged by 10 items on a Likert scale between 1 (“strongly disagree”) and 6 (“strongly agree”). The measure is divided in two subscales, five items each: Working Excessively (*WE*, α = 0.68–0.78, e.g., “I spend more time working than on socializing with friends, on hobbies, or on leisure activities”), and Working Compulsively (*WC*, α = 0.73–0.78, e.g., “I feel obliged to work hard, even when it is not enjoyable”). In our study, the measure received good reliability (see Table 2).
- *Bergen Work Addiction Scale* (BWAS; Andreassen et al., 2012) was gauged by seven items on a Likert scale between 1 (“never”) and 6 (“always”), with good reliability (α = 0.80–0.84, e.g., “spent much more time working than initially intended?”). In our study, the measure received good reliability (see Table 2).
- *Heavy-Work Investment* (HWI) was gauged by 10 items on a Likert scale between 1 (“strongly disagree”) and 6 (“strongly agree”). The original measure is based on Brown and Leigh's (1996) paper and was named “effort” in work. The measure is divided into two subscales, five items each: Time Commitment (*TC*, α = 0.82–0.86, e.g., “Few of my peers put in more hours weekly than I do”), and Work Intensity (*WI*, α = 0.82–0.83, e.g., “When I work, I really exert myself to the fullest”). However, it was recently conceived as a new concept: Heavy-Work Investment (Snir and Harpaz, 2012, 2015). Meaning, a heavy-work investor must be rated high on both—time and effort—invested in the job. In our study, the measure received good reliability (see Table 2).
  Table 2 presents the reliability coefficients, ranges, means and standard deviations for all of the variables.
- *Common-method bias*. In order to test for common-method bias (CMB), we employed Harman's single-factor test (see Podsakoff et al., 2003). The single-factor explained 32.8% of the variance in the Israeli sample and 29.6% in the Romanian sample, and as such is not considered to have CMB problems (criterion for CMB problems is *R*^2^ > 50%).

**Table 2:** Reliability coefficients, ranges, means, standard deviations, for both samples.

**Variable**	**Subscale**	**Israeli sample**				**Romanian sample**			
		**α**	**Range**	***M***	***SD***	**α**	**Range**	***M***	***SD***
BWAS^a^	–	–	–	–	–	0.81	1–6	2.85	0.94
DUWAS^b^	WC^c^	–	–	–	–	0.73	1–6	3.15	0.96
	WE^d^	–	–	–	–	0.76	1–6	3.44	1.02
	Total	–	–	–	–	0.85	1–6	3.30	0.92
HWI^e^	TC^f^	–	–	–	–	0.83	1–6	3.41	1.08
	WI^g^	–	–	–	–	0.92	1–6	4.49	0.99
	Total	–	–	–	–	0.87	1–6	3.95	0.86
WFBS^h^	Cognitive^i^	0.86	1.3–6	3.98	1.06	0.85	1–6	3.71	0.99
	Emotional	0.56	1.1–5.6	3.27	0.76	0.58	1–6	3.18	0.77
	Instrumental	0.75	1.1–6	3.86	0.96	0.77	1–6	3.74	0.90
	Time	0.74	1.4–5.7	3.38	0.82	0.76	1.1–6	3.26	0.82
	Effort	0.76	1.6–5.7	4.04	0.81	0.75	1–6	3.83	0.73
	Total	0.85	1.6–5.7	3.71	0.74	0.86	1.2–6	3.54	0.72

a *BWAS, Bergen Work Addiction Scale*.

b *DUWAS, Dutch Work Addiction Scale*.

c *WC, Working Compulsively*.

d *WE, Working Excessively*.

e *HWI, Heavy-Work Investment*.

f *TC, Time Commitment*.

g *WI, Working Intensely*.

h *WFBS, Workaholism Facet-Based Scale*.

i *The WBFS' elements of each facet*.

### Procedure

The full survey was delivered in two manners: (1) online internet questionnaires and (2) hard-copy questionnaires. The majority of the Israeli sample was derived from the internet source (63%) as opposed to the hard-copy one (37%). The Romanian sample was solely derived from the internet source. The data were analyzed using SPSS (v. 22) and AMOS (v. 22) software packages.

## Results

### About the analyses

In order to test our hypotheses, we employed two different analyses methods: (1) factor analysis and (2) similarity structure analysis. In the next section we explain their purposes and differences. We did so aiming to find the most elegant data representation in accordance with the suggested mapping sentence.

Factor analysis (FA) is a variable-directed multivariate statistical technique, which depends on an identified statistical model. FA explains the covariance and/or correlation structures among the measured variables. Another purpose is to develop a new set of uncorrelated variables, with the aim of giving a better understanding of the data, using the smallest sets of variables. Meaning, it strives to be more parsimonious (Spearman, 1904).

Similarity structure analysis or smallest space analysis (SSA) is a non-metric case of multidimensional scaling (MDS) used in the facet theory (Gaul et al., 2009). It was developed by Guttman (1968) later than MDS. Its aim is to depict the data in the smallest number of dimensions available (Bloombaum, 1970). Like MDS, SSA is a form of non-linear dimensionality reduction analysis. It is a mathematical technique that allows mapping of distances between points in dimensional/geometric spaces. Points are, in fact, the variables measured in a data set. Most common, and most useful, is a two-dimensional mapping of the points (see Cox and Cox, 2001), which may be visualized in a plot. The points can be seen as distant (dissimilar) or close (similar) to each other. When a similarity between two items is high, the distance between the geometric points representing them (e.g., in a diagram) is relatively small. Conversely, when the similarity between two items is low (i.e., dissimilarity), the distance between their points should be relatively large (Elizur, 1984). MDS can also be used as cluster analysis for grouping observations (Young and Hamer, 2013). SSA is less restrictive than FA, and such an unrestrictive approach could potentially reveal “insights that classical factor analytic techniques seem to have hidden” (Sternberg, 1984, p. xii). SSA produces two goodness-of-fit indices, namely *Coefficient of Alienation* (COA) and *Regionality Index* (also known as Separation Index). COA is the degree to which the geometric distances between the points on the dimensional map reliably reflect their interrelations, meaning, how the algorithm had to make concessions in order to display them. COA ranges between 0 and 1, and the lower the coefficient, the better the fit (Friedman, 2008). Regionality Index evaluates the extent to which the obtained empiric model reflects the assumed content facets, that is to say, the congruence between the theoretical model and the spatial dispersion (corresponding distances) of the empiric data (Friedman, 2008). This index ranges between 0 and 1; the higher the value, the better the fit. In this paper we used the ALSCAL algorithm of the SPSS software and the SMACOF algorithm of the R software. As such, there is one widely accepted fit index—*stress*, although the way in which it is computed differs from one algorithm to the other (for further reading, see Jacoby, 2012).

SSA and FA share a common purpose—reducing the number of variables/items by making parsimonious groupings (Maslovaty et al., 2001). However, there are several main differences between the methods (Guttman, 1982), such as: (1) SSA is more flexible in describing the relationships among variables; (2) SSA represents domains in fewer dimensions (parsimony); (3) FA's technique relies on strict assumptions of linearity, while SSA allows for possible non-linear relationships; (4) Similarity coefficients are not adjusted for reliability; (5) SSA results may be easier to represent in a visual geometric form; (6) For SSA results to be meaningful, a large sample size is not critical.

### Exploratory factor analysis (EFA)

We performed EFA (rather than confirmatory FA, see Sternberg, 1984) to see how the items converge with a linear modulation. The analyses found 5 and 4 different factors (Varimax rotation) for the Israeli (*R*^2^ = 0.65) and Romanian (*R*^2^ = 0.59) samples, respectively. We also performed forced analyses so that the items would converge on a single-factor (*R*^2^ = 0.33 and *R*^2^ = 0.36 for Israeli and Romanian samples, accordingly), in order to compare the results to a factorized solution as opposed to a single-factor one. All of the solutions, as expected, however, had poor model fit (Byrne, 2010). The *5-factor* solution (Israeli sample): $\chi_{\left(110\right)}^{2}$ = 279.38, *p* = 0.000, χ^2^/df = 2.54, SRMR = 0.08, CFI = 0.84, NFI = 0.77, GFI = 0.84, ECVI = 2.21, RMSEA (90% CI) = 0.10 (0.08–0.11), *p* = 0.000. The *5-factor* solution (Romanian sample): χ^2^_(129)_ = 1,519.29, *p* = 0.000, χ^2^/df = 11.78, SRMR = 0.08, CFI = 0.82, NFI = 0.81, GFI = 0.85, ECVI = 1.43, RMSEA (90% CI) = 0.10 (0.09–0.10), *p* = 0.000. The *single-factor* solution (Israeli sample): χ^2^_(135)_ = 495.75, *p* = 0.000, χ^2^/df = 3.67, SRMR = 0.11, CFI = 0.68, NFI = 0.61, GFI = 0.73, ECVI = 3.44, RMSEA (90% CI) = 0.13 (0.12–0.14), *p* = 0.000. The *single-factor* solution (Romanian sample): χ^2^_(135)_ = 2,686.62, *p* = 0.000, χ^2^/df = 19.90, SRMR = 0.10, CFI = 0.67, NFI = 0.66, GFI = 0.75, ECVI = 2.47, RMSEA (90% CI) = 0.13 (0.12–0.13), *p* = 0.000. The factor analyses and loadings are presented in Table 3.

**Table 3:** Exploratory factor analyses loadings and results for the WFBS.

	**Israeli sample (*N* = 166)^a^**						**Romanian sample (*N* = 1,117)^b^**				
	**1**	**2**	**3**	**4**	**5**	**Single^c^**	**1**	**2**	**3**	**4**	**Single^c^**
Item 1	0.82					0.76	0.63				0.65
Item 7	0.85					0.72	0.85				0.72
Item 13	0.71					0.82	0.74				0.78
Item 2	0.68					0.68			0.61		0.61
Item 8	0.79					0.74	0.81				0.75
Item 14		0.60				0.51			0.49		0.59
Item 15		0.57						0.77			0.50
Item 11		0.64				0.65		0.46			0.65
Item 17		0.68				0.66		0.55			0.59
Item 6			0.77			0.40			0.78		0.42
Item 12			0.59			0.76			0.75		0.64
Item 18			0.70			0.63			0.66		0.63
Item 9				0.67		0.46		0.58			0.52
Item 10				0.78		0.47		0.73			0.56
Item 16				0.66		0.41		0.77			0.55
Item 3 (*R*)^d^					0.80					0.82	
Item 4 (*R*)					0.75					0.82	
Item 5					−0.55	0.40			0.46		0.64
Eigenvalue	3.55	2.32	2.03	1.98	1.86	5.90	3.11	3.05	2.98	1.65	6.17
*R*^2^	0.20	0.13	0.11	0.11	0.10	0.33	0.17	0.17	0.16	0.09	0.34
α coefficient^e^	0.88	0.67	0.72	0.61	0.64	0.87	0.86	0.79	0.78	0.66	0.90

*Loadings are based on Varimax rotation in Principal Axial Factoring (PAF) method. Factor loadings with a value of 0.40 or higher (in absolute value) were retained*.

a *For Israeli sample KMO = 0.83, Bartlett's χ^2^_(153)_ = 1,228.83, p = 0.000*.

b *For Romanian sample KMO = 0.89, Bartlett's χ^2^_(153)_ = 7,963.51, p = 0.000*.

c *Single, single-factor solution*.

d *R, reverse-coded items*.

e *Cronbach's Alpha coefficient*.

FA identified only the cognitive (a1, except item 14 for both samples) and emotional (a2, except item 5 in the Israeli sample) elements of the modalities facet, and time (b1) element of the resources facet, in both samples (see Table 3). FA also identified the effort (b2) element in the Romanian sample. Moreover, the third factor (in both samples) is a combination of both elements—instrumental (a3) and effort (b2), and not a pure element. However, we had two exceptional items (5 and 14) which did not converge logically well with the factors' solution. Nevertheless, as will be shown below, they do converge very well in the similarity structure analysis (SSA).

In addition, we performed a FA for the other measures in the second phase of the research (with the Romanian sample). The results are presented in Table 4.

**Table 4:** Exploratory factor analyses loadings and results for other measures, for Romanian sample (*N* = 1,117).

	**DUWAS**		**BWAS**	**HWI**	
	**1**	**2**	**1**	**1**	**2**
Item 1	0.84		0.67		0.58
Item 2	0.75		0.65		0.81
Item 3		0.59	0.68		0.83
Item 4		0.81	0.74		0.74
Item 5		0.78	0.69		0.82
Item 6	0.73		0.68	0.84	
Item 7	0.67		0.71	0.86	
Item 8	0.68		–	0.85	
Item 9		0.64	–	0.86	
Item 10		0.68	–	0.85	
Eigenvalue	3.01	2.72	3.29	3.80	3.03
*R*^2^	0.31	0.27	0.47	0.38	0.30
KMO	0.86		0.83	0.88	

*Loadings are based on Varimax rotation in Principal Axial Factoring (PAF) method. Factor loadings with a value of 0.40 or higher (in absolute value) were retained. (1) Items 1–5, Working Compulsively; items 6–10, Working Excessively (Schaufeli et al., 2009). (2) Andreassen et al., 2012. (3) Items 1–5, Time Commitment; items 6–10, Working Intensely (Brown and Leigh, 1996). (4) This row represents the number of factors derived from the analyses*.

As can be seen, the DUWAS, for example, did not converge as its authors intended, but the BWAS and the HWI measures did align with their intended use.

### Similarity structure analysis (SSA)

As FA showed partial concordance with our mapping sentence and facets, we proceeded to perform SSA analyses. SSA is an intrinsic data analysis technique with an emphasis on looking at regions in the geometric space of variables rather than at coordinate systems (Levy, 2005). We, however, wish to elaborate on two well-known algorithms for SSA analyses: (1) ALSCAL, and (2) SMACOF.

ALSCAL (Alternating Least Squares SCALing, see Takane et al., 1977) is an older algorithm than SMACOF (Scaling by MAjorizing a COmplicated Function, see de Leeuw and Heiser, 1980; Young and Hamer, 1987, 2013). The latter improved upon the former in three main fashions: (1) speed, elegance and simplicity, (2) it is based on distances (including negatives) and not squared-distances (Young and Hamer, 1987, 2013), (3) minimizing the Stress function (see de Leeuw and Mair, 2009). Another advantage is that the SMACOF algorithm can only be used in the R software package (version 2.7.0 and later) which is a free open-source program and “the functions available in R implement many state-of-the-art statistical procedures, and the graphics are better than those available in any other software package” (Jacoby, 2012). SMACOF in R can be implemented in many fields and practices, such as: social sciences, individual differences, geography, 2D and 3D graphical presentations, rectangular matrices, quadratic surfaces, using metric and non-metric data, and more (de Leeuw and Mair, 2009).

**Figure 3:**
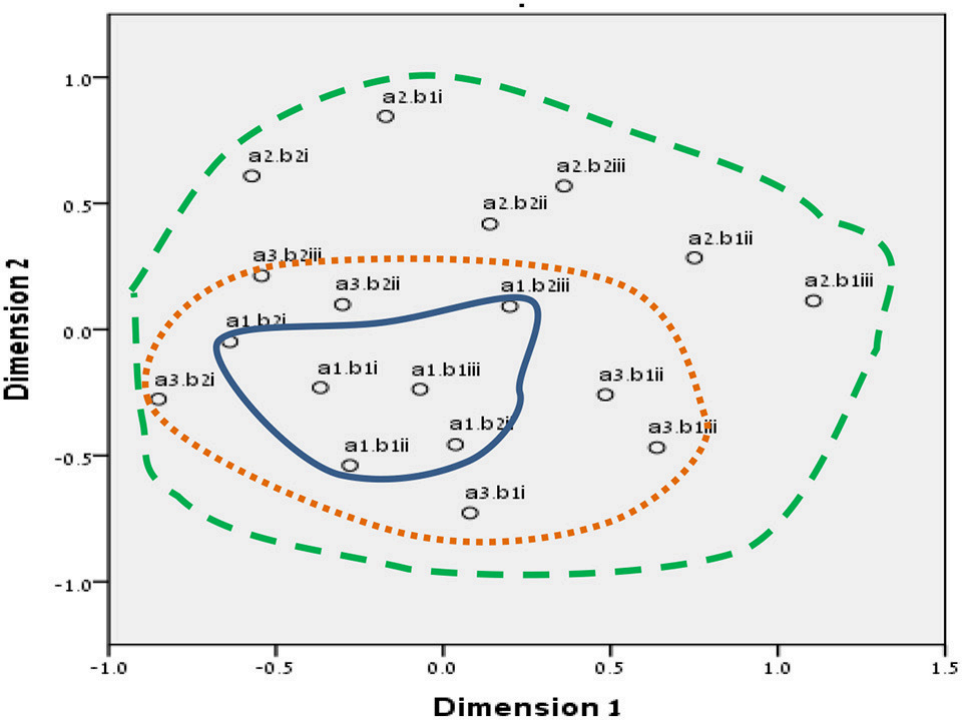
SSA common space diagram for modalities facet (A) of the WFBS, Israel. Solid line, Cognitive (a1); dotted, Instrumental (a3); dashed, Emotional (a2).

**Figure 4:**
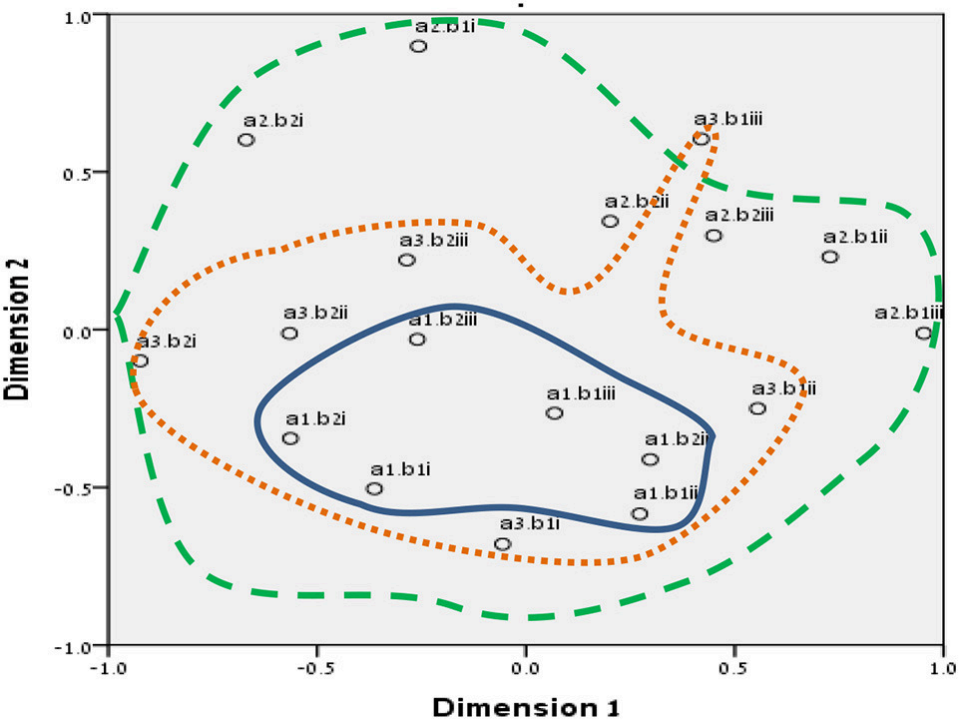
SSA common space diagram for modalities facet (A) of the WFBS, Romania. Solid line, Cognitive (a1); dotted, Instrumental (a3); dashed, Emotional (a2).

**Figure 5:**
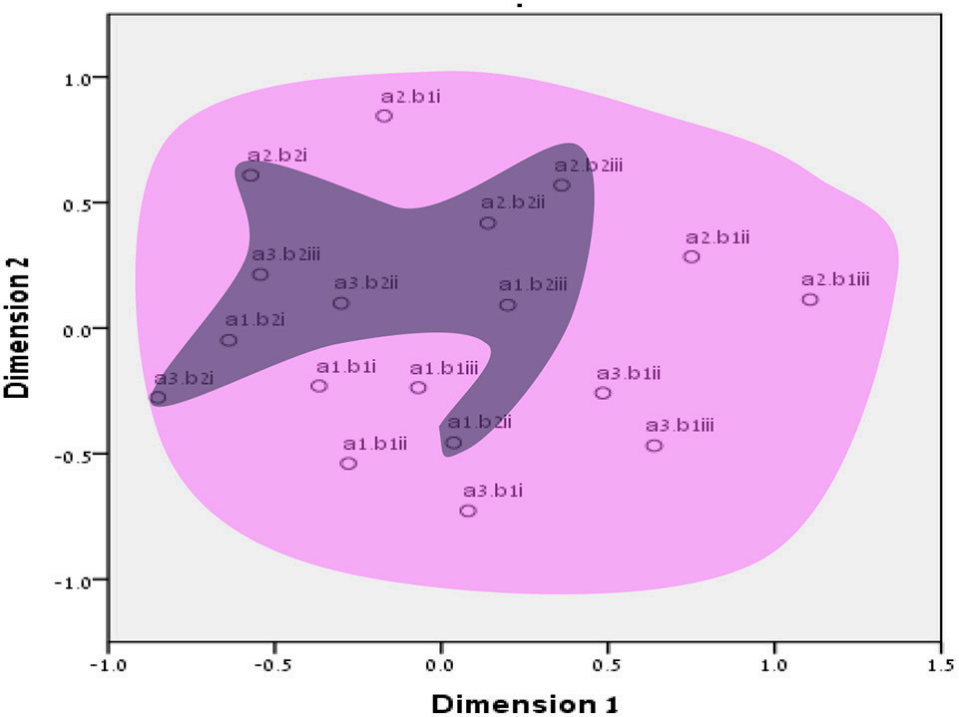
SSA common space diagram for resources facet (B) of the WFBS, Israel. Light-filled surface, Time (b1); Dark-filled surface, Effort (b2).

**Figure 6:**
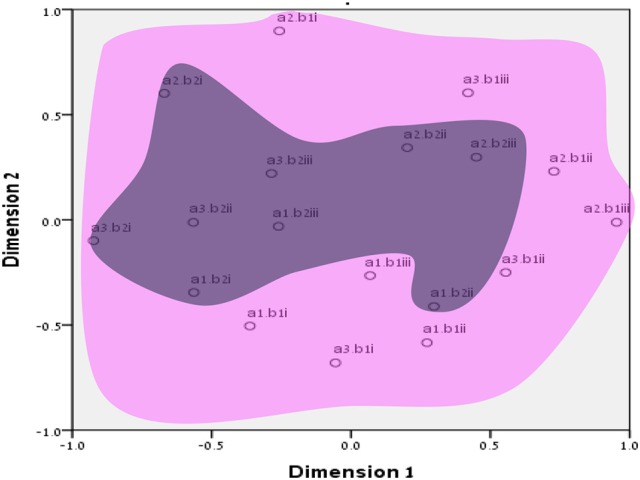
SSA common space diagram for resources facet (B) of the WFBS, Romania. Light-filled surface, Time (b1); Dark-filled surface, Effort (b2).

**Figure 7:**
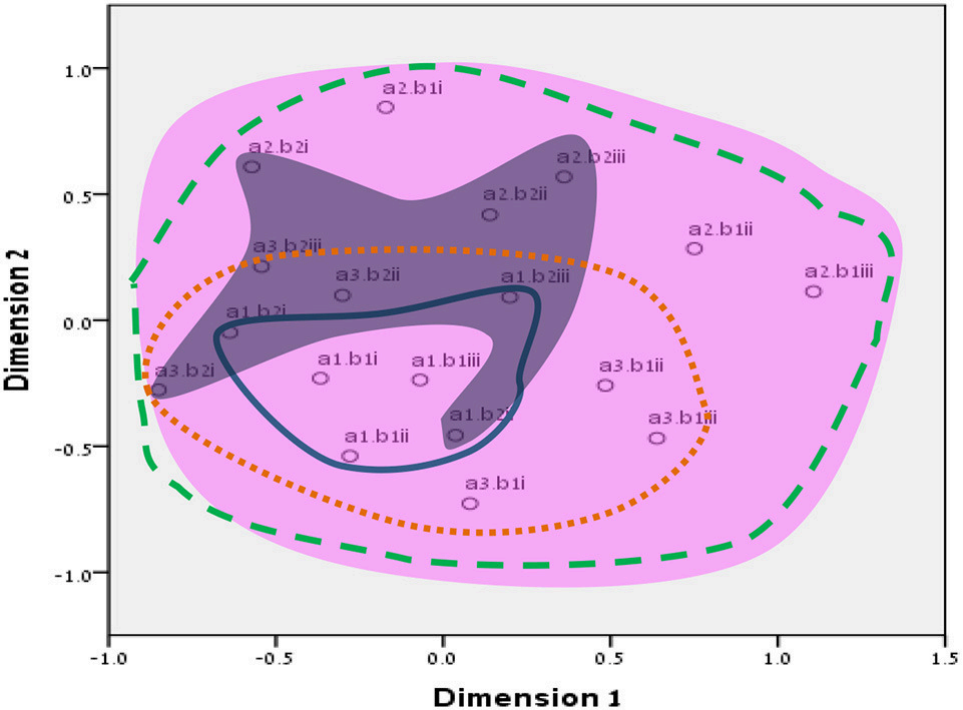
Total SSA common space diagram for the WFBS, Israel. Solid line, Cognitive (a1); dotted, Instrumental (a3); dashed, Emotional (a2). Light-filled surface, Time (b1); Dark-filled surface, Effort (b2). Stress = 0.05 (SMACOF).

**Figure 8:**
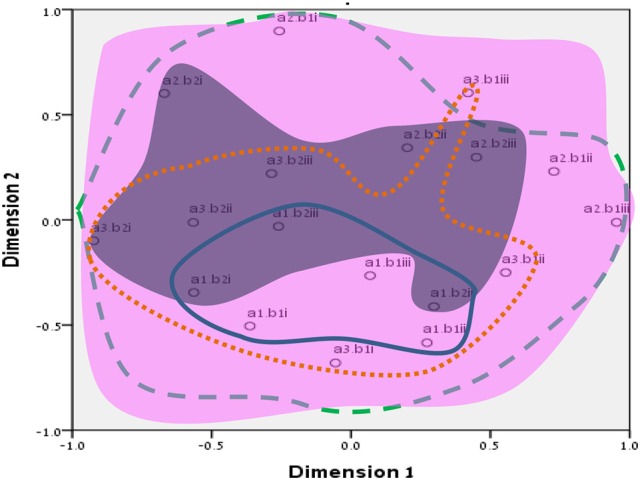
Total SSA common space diagram for the WFBS, Romania. Solid line, Cognitive (a1); dotted, Instrumental (a3); dashed, Emotional (a2). Light-filled surface, Time (b1); Dark-filled surface, Effort (b2). Stress; 0.05 (SMACOF).

We chose to run SSA with the two multidimensional scaling algorithms ALSCAL (in SPSS vs. 22) and SMACOF (in R v. 3.4.1). The divergence and convergence of the items in both methods resembled our mapping sentence and hypotheses more accurately. The results are shown in Figures 3–8 for the Israeli and Romanian samples, accordingly, and both analyses indicated good and almost-identical fit for both samples (see Kruskal, 1964; Dugard et al., 2010; Jacoby, 2012) Stress = 0.05 and 0.06 (for SMACOF and ALSCAL in the Israeli sample, respectively) and Stress = 0.04 and 0.05 (for SMACOF and ALSCAL in the Romanian sample, respectively). This also shows: (a) the superiority of SSA over FA (in fit indices and intensifying the structure of the mapping sentence), (b) SMACOF algorithm produced better fit than the ALSCAL. However, it can clearly be seen that the disparity of the items in the Romanian sample is far larger than in the Israeli one, regardless of the almost-identical fit indices. This implies that it is not mandatory for the items to be in very close proximity to each other in order to be meaningful and retain their facet structure.

In order to encompass our statistical options, after having employed EFA and SSA, we also used a CFA analysis for the SMACOF results (in AMOS v. 22), as the fit of the model was greater (see Appendix C). As can be seen in the analyses, for both Israeli and Romanian samples, SSA is still superior to both FA methods.

**Figure 9:**
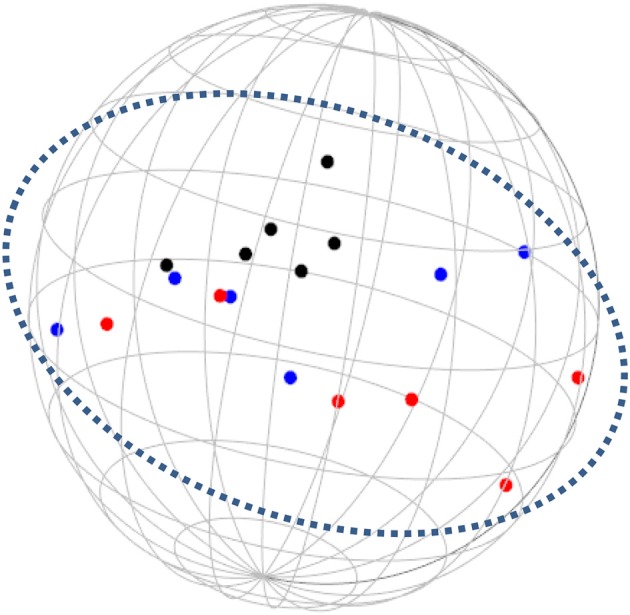
Total 3D SSA common space diagram (vertical) for the WFBS, Israel.

**Figure 10:**
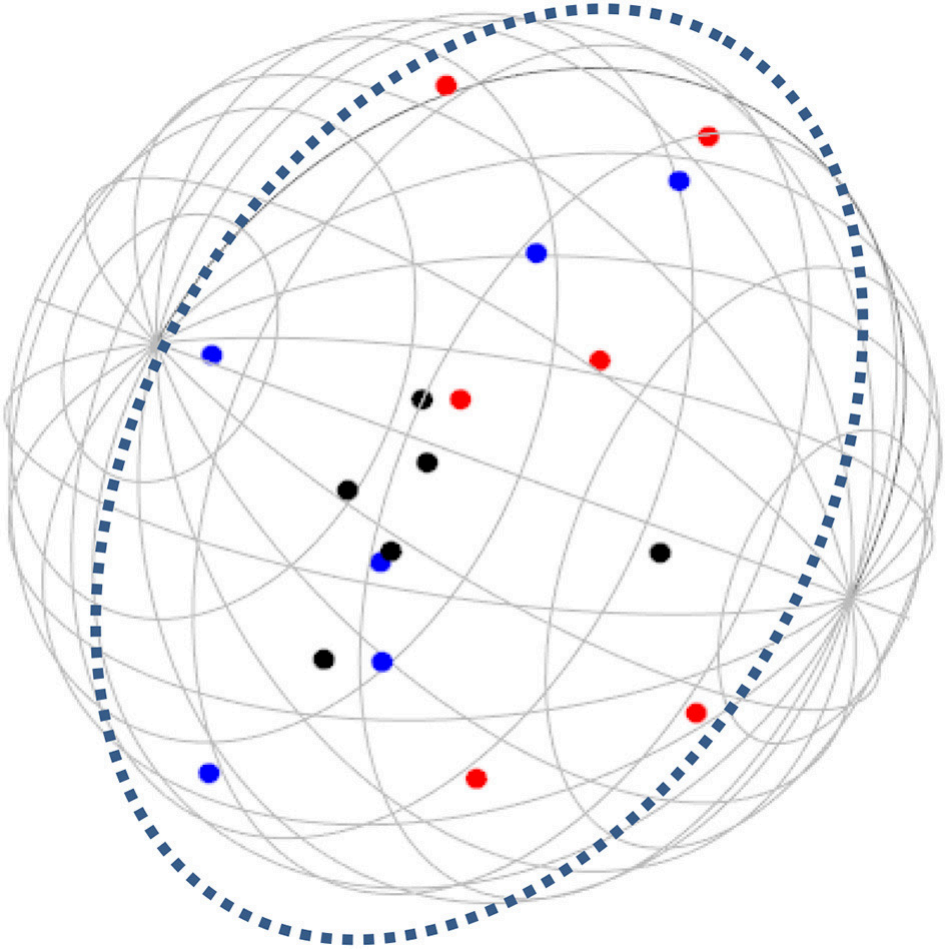
Total 3D SSA common space diagram (horizontal) for the WFBS, Israel.

**Figure 11:**
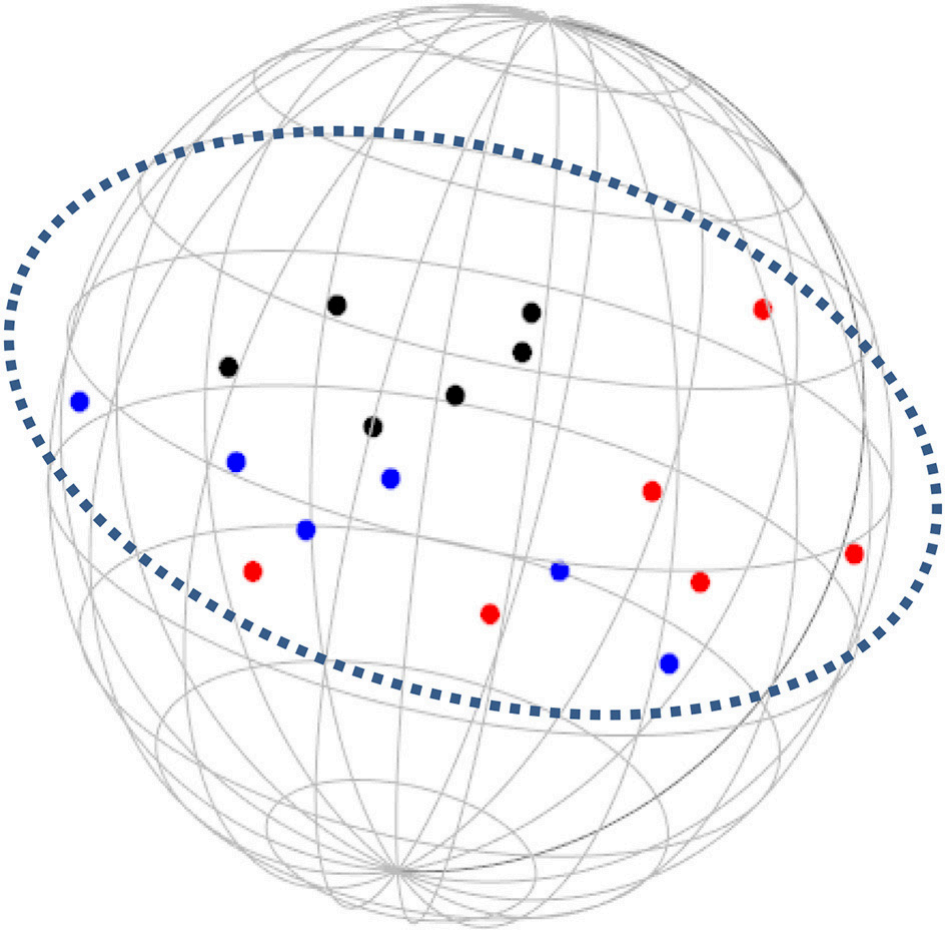
Total 3D SSA common space diagram (vertical) for the WFBS, Romania.

**Figure 12:**
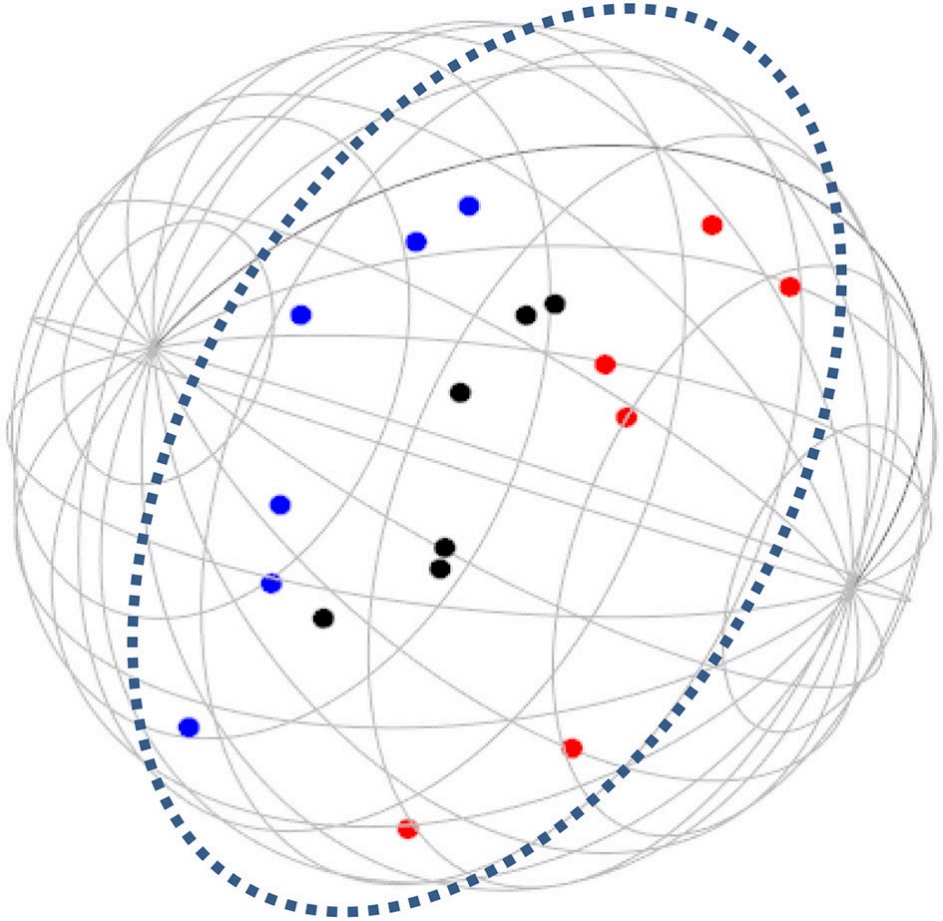
Total 3D SSA common space diagram (horizontal) for the WFBS, Romania.

Furthermore, the SMACOF results converged on a three-dimensional space, meaning the data is multidimensional (not fitting for FA), and must also be visualized in a 3D diagram. Using the R software, we produced 3D spheres with the SMACOF coordinates (see Figures 9–12).

### Regarding our hypotheses

1. Workaholism can be classified according to its modalities facet (cognitive, emotional, and instrumental) and the workaholism resources facet (time, effort). Meaning, the empirical results reflect the components of workaholism, as defined in the mapping sentence. Each facet and its elements occupy a distinct region.
2. Observing Figures 3, 4 for the Israeli and Romanian samples, respectively (the structure of facet A, workaholism modalities), three circular regions can be clearly distinguished—cognitive items in the center, instrumental items next in the second circle, and emotional items in the peripheral region. This facet structure (organizing the items from the center to the periphery) is called a modulating facet. As such, our hypothesis for a polarizing facet was not supported in this regard. The results showed a modulating structure with a great fit.
3. Observing Figures 5, 6 for the Israeli and Romanian samples, respectively (the structure of facet B, workaholism resources), two circular regions can be clearly distinguished—effort items in the center, and time items in the peripheral region. This facet structure (organizing the items from the center to the periphery) is called a modulating facet. Thus, our hypothesis for a modulating structure was indeed supported, but the elements were laid out in reverse order to our hypothesis (which assumed that the time items would be central, and the effort items—peripheral). This structure had a great fit as well.
4. Observing Figures 9–12 for the Israeli and Romanian samples (the total 3D structure of the SSA, both facets), we can clearly see it is not a radex configuration, but rather an oblate *ellipsoid* (which might be the outcome of two combined modulating facets). In which case, the structure in the Romanian sample was tighter, more oblate than the Israeli.

Lastly, in order to see the associations among the various variables and their subscales (only for the Romanian sample), a Pearson correlation matrix was formed, as shown in Table 5.

**Table 5:** Pearson correlation matrix, for Romanian sample (*N* = 1,117).

		**1**	**2**	**3**	**4**	**5**	**6**	**7**	**8**	**9**	**10**	**11**	**12**
	BWAS												
**1**	WC	0.67											
**2**	WE	0.65	0.74										
**3**	DUWAS	0.71	0.92	0.93									
**4**	TC	0.55	0.55	0.62	0.63								
**5**	WI	0.20	0.31	0.39	0.37	0.38							
**6**	HWI	0.47	0.52	0.61	0.61	0.84	0.81						
**7**	Cognitive	0.53	0.53	0.56	0.59	0.52	0.41	0.56					
**8**	Emotional	0.31	0.39	0.25	0.34	0.26	0.15	0.25	0.37				
**9**	Instrumental	0.52	0.50	0.56	0.57	0.60	0.45	0.64	0.67	0.35			
**10**	Time	0.62	0.62	0.59	0.65	0.59	0.31	0.55	0.82	0.62	0.79		
**11**	Effort	0.43	0.47	0.48	0.51	0.48	0.49	0.59	0.78	0.62	0.79	0.71	
**12**	WFBS	0.57	0.60	0.58	0.63	0.58	0.43	0.61	0.87	0.67	0.85	0.93	0.91

*All of the correlations are statistically significant at p < 0.001. BWAS, Bergen Work Addiction Scale; DUWAS, Dutch Work Addiction Scale; WE, Working Excessively; WC, Working Compulsively; HWI, Heavy-Work Investment; TC, Time Commitment; WI, Working Intensely; WFBS, Workaholism Facet-Based Scale*.

As seen in Table 5, the measures are not foreign to each other and have good correlations with one another. Regarding the WFBS subscales, they also correlated highly with each other, although the emotional subscale had the lowest associations of them all.

## Discussion

The main goal of this paper was to attempt and clear some of the confusion surrounding workaholism (see Clark et al., 2016) in a two-phase research. In light of these operationalization and conceptual difficulties, we embraced the Facet Theory approach (Guttman, 1954, 1957).

In the first phase, we formulated a theoretical definitional framework for workaholism. The empirical results supported the definitional framework suggested for the workaholism domain. The components of workaholism, as defined in the mapping sentence, were indeed reflected, and distinct regions for each of the facets and their elements (A Modalities: cognitive, emotional, and instrumental; B Resources: time and effort) could clearly be distinguished.

In regard to facet A (modalities), as opposed to our hypothesis for a polarizing structure, three circular regions were distinguished—cognitive items in the center, instrumental items next in the second circle, and emotional items in the peripheral region. Meaning, this facet is a modulating one. As workaholism is defined as an uncontrollable inner drive, it is not exclusively instrumental/physical, but it is first reflected cognitively. Meaning, the employee may think about work even when absent from it. Usually, thought precedes action, and this explains the central regionality of the cognitive element (see also Harpaz and Snir, 2016).

The emotional element, though existing in most of the measures of workaholism (e.g., Spence and Robbins, 1992; Robinson, 1998; Schaufeli et al., 2009; Andreassen et al., 2012), was not fully theoretically defined until the current study, a far as we know. As can be seen in Figures 3, 4 (for the Israeli and Romanian samples, accordingly) the emotional element is the most peripheral and the disparity of its points is far larger than any other element. While there is no denying what “*thinking* about work” and “*doing* work” mean, “*feeling* about work” is more obscure. Emotions about work may be positive or negative (based on interpretation) and might be culturally-dependent as well, while “doing” and “thinking” about work are less ambiguous.

**Figure 13:**
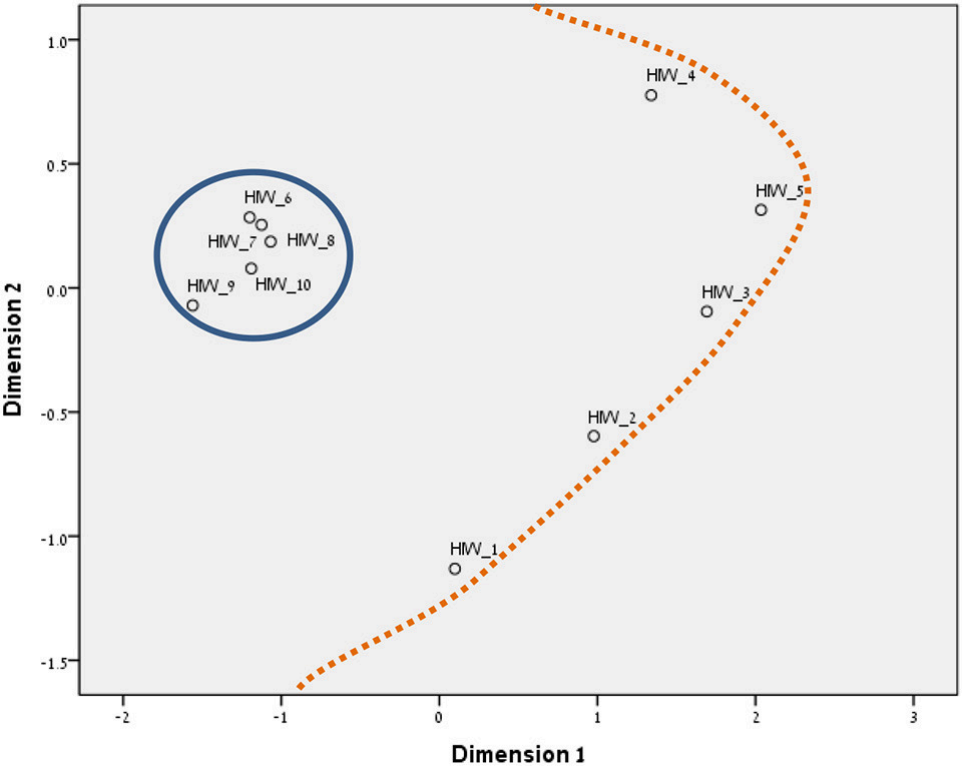
SSA common space diagram for the HWI measure, Romania. Stress = 0.04; Solid line, Working Intensely; dotted line, Time commitment.

In regard to facet B (resources), as opposed to our hypothesis that the time items would be central, and the effort items—peripheral, the effort items were placed in the central circle and the time items in the peripheral circle of the modulating structure. Support for this modulation (albeit reversed) can be seen in the second phase SSA results regarding HWI (see Figure 13); the effort (working intensely) items are very much converged, as opposed to the time commitment items. Effort investment may be accompanied by time commitment, but not necessarily vice versa. Like the presenteeism phenomenon, when an individual invests time in work, an investment of effort will not always follow (e.g., Pseudo-Heavy Work Investment, see Astakhova and Hogue, 2015. Low-Heavy Work Investment, see Rabenu and Aharoni-Goldenberg, in press). Another plausible explanation may be nested in methodological reasoning: as stated before, the time aspect is more generally agreed upon due to its universal measurement scale (i.e., seconds, minutes, working hours, formal work break, etc.). We, therefore, tried to encompass time's entire scale range, which resulted in more scattered items. However, since effort is less agreed upon, we were trying to be careful and stick to its terminology, which led us to generate items that were semantically closer to each other, as opposed to the items in the time element. This may have been the reason behind the higher similarity of the effort items.

Our hypothesis concerning the total structure of workaholism (radex) was thus not supported, due to the different nature of the facets attained in the analyses, since the two facets, modalities (A) and resources (B), were both modulating. Such a combination of two modulating facets may be quite confusing to the observer (dual circular layout; see end of results section); however, the possible conceptual combination is much clearer and interesting. In a circumflex structure of one modulating facet, the proximity between the elements in the circular layout hints at a functional dependence between close items, whether they differ qualitatively or quantitatively (Friedman, 2008). Ergo, in our results of two modulating facets, the core of the cognitive element (a1) is comprised of the time (b1) items while the effort (b2) items are scattered in the peripheral area of the cognitive circle. This occurred only in the Israeli sample, and a possible explanation is the occupational differences between the two samples. In Israel, there were statistically significant χ^2^ (2, *N* = 1,238) = 23.98, *p* = 0.000, *r*_c_ = 0.14) more high-tech workers (27.1%) in comparison to Romania (13.7%), while the Romanian sample was more low-tech oriented (28.3%) than the Israeli one (16.9%), although the samples did not differ in the services industry (56 and 58% for Israelis and Romanians, accordingly). Especially for the high-tech industry “… long hours are the norm. Those present are assumed to be working elsewhere… others will do so in their minds and—a few would report—even their dreams” (Kunda, 2009, p. 3). This demonstrates that high-tech is vastly characterized by high cognitive investment, throughout the entire day cycle. In addition, even in correlational terms (see Table 5), the relationship between the cognitive element and the time element was stronger than the association between the cognitive and effort elements.

Furthermore, employees working longer hours may be idolized as “heroes” and displayed as role models (Shimazu et al., 2015). Such a reward system may promote workaholic behaviors by setting fewer limitations on excessive work routines (Mazzetti et al., 2014). Similar organizational cultures, those promoting long working hours, may become triggers which may activate the drive to work disposition (Harpaz and Snir, 2016). However, effort may be more internal and less outwardly visible than time. For example, many managers observe the amount of time that employees are present at work as an indication of their performance, especially when it is difficult or complicated to assess the output (Tziner and Rabenu, 2011). In addition to the differences between industries, they may be cultural as well. For example, in our findings (see Figures 3, 4), it can be seen that item no. 17 of WBFS [“… work all the time, even on breaks (e.g., lunch breaks, smoking breaks, etc.)”] converged well with the instrumental element (a3) in the Israeli sample, according to the mapping sentence. However, it was more proximal to the emotional (a2) one in the Romanian sample (where it should not belong). It is even more interesting to notice that item 17 is proximal to items 10 and 16 which both reflect “enthusiasm about investing effort in work.” As Israel is known to have an overworking job culture, working much more hours than the OECD's average (OECD, 2013), investing time in working (even on breaks) may seem normative. However, in Romania, one must probably have high drive for work (i.e., being a workaholic) in order to be enthusiastic about pouring effort into it on breaks.

Nonetheless, comparing the main results from Israel and Romania, they were relatively similar, despite the Israeli one had less participants (*N* = 166) than the Romanian (*N* = 1,117). As Israel is already considered a highly overworking country, all of the WBFS subscales (in terms of means) were higher than in the Romanian sample (see Table 2). The discussion about cultural differences makes cross-cultural, in general, and Romania, in particular, an interesting field to further research work-related topics.

**Figure 14:**
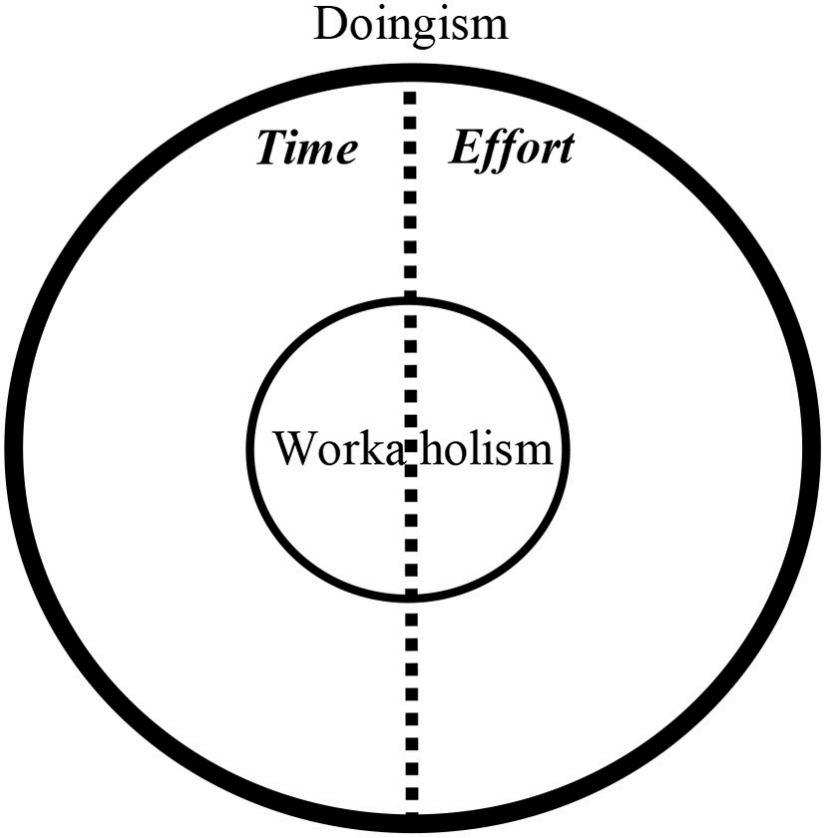
Suggested conceptual map for doingism and workaholism. It is important to note that since the year 2000, the research about time investment has been vast (e.g., Dembe et al., 2005; Caruso, 2006; Suzuki et al., 2017), but is lacking in regards to the investment of effort (to the best of our knowledge; see Green, 2008), the proportions of the inner the elements in the figure may vary.

Since the mapping sentence was supported, and according to the results that the cognitive and effort elements were the most central of the two facets (modalities and resources, accordingly), it may be argued that workaholism is a private case of a phenomenon we may call “Doingism.” Doingism is a portmanteau composed of the words “doing” and “alcoholism” (on a similar notion that workaholism is composed of “work” and “alcoholism”). Its definition resembles the one defined in the mapping sentence of workaholism in the current paper, except that it is more general and not workplace-specific. Meaning, a workaholic is driven to heavily invest in the work itself (i.e., effort and time), while the “doingist” is driven to heavily invest in and out of the job (e.g., at home, volunteering, etc.). As such, by Set Theory terms, workaholism is a subset of doingism (i.e., all the elements of workaholism are also elements of doingism); workaholism ⊆ doingism. The doingist is driven to “do” in general, not only at work, while the workaholic is driven to “do work” exclusively. That is the reason workaholism is a private case of doingism (in the workplace). Depending on contextual differences, doingism may be translated into workaholism since working is usually valued (see also Shimazu et al., 2015). See Figure 14 for a conceptual map.

**Figure 15:**
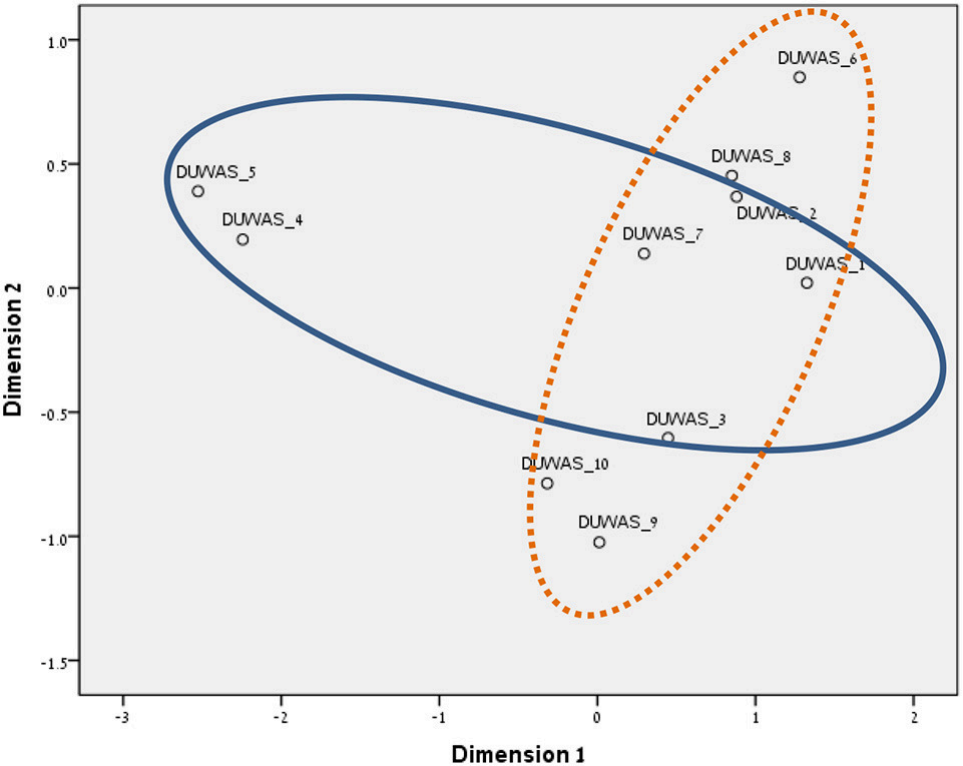
SSA common space diagram for the DUWAS measure, Romania. Stress = 0.06; Solid line, Working Compulsively; dotted line, Working Excessively.

Regarding the comparisons among the different measures in our study (i.e., BWAS; Andreassen et al., 2012, DUWAS; Schaufeli et al., 2009, HWI; Snir and Harpaz, 2012, 2015), although all of them had good reliability and model fit indices from FA and SSA (not including BWAS which had no subscales), there were still some oddities in a few findings. As can be seen in Table 5, the DUWAS items do not converge in FA as its authors intended, and did not do as well as in SSA (see Figure 15). This may be a byproduct of “arbitrariness” with producing the measure's items, as opposed to doing so by a formal conceptual framework and a Cartesian multiplication of theoretically based item composition (see also Clark et al., 2016). In addition, regarding our new measure, FA (see Table 5) recognized our elements only partially [modalities (A)—cognitive (a1) and emotional (a2), resources (B)—time (b1) and effort (b2)], but even so, most of the factors were “contaminated” with items from other elements (e.g., time with effort items, etc). While FA needed 4–5 (sometimes contaminated) factors for its solution, SSA resulted in a much clearer and more elegant picture of the facets consistent with the mapping sentence. Only two dimensions (modalities and resources) were needed for this solution, and as such it is more parsimonious. Thus, SSA has an advantage in describing the concept in the fewest parameters possible, an approach embraced in current scientific notions. This stresses the necessity of using the facet theory methodological framework.

### Future research

We recommend replicating the current study in more cross-cultural contexts. As already shown in the paper, we found interesting differences between the Israeli and the Romanian samples, which lead us to believe that such comparisons would benefit the literature and broaden the generalizability of the results.

We also think it would be highly interesting to test known research models regarding workaholism with our new measure (WFBS) and see how the outcomes differ. Perhaps WBFS will shed new light on former findings, a paraphrase on “new wines in old bottles.” In addition, because it is a Cartesian product, the WBFS can be used as 18-, 12-, and 6-item scales. We also tested the reliability for each derivative and the results were adequate. We thus encourage researchers to use the measure differently with same models, in order to see if and how the results vary.

There should be more validating papers accounting for WFBS and constructs which have known associations with workaholism (convergent validity) or are unrelated to it (discriminant validity). Our measure may enable to gauge the nature of workaholism in which individuals might feel *pushed* (driven) to the act of working; while on the other hand, job engagement or harmonic passion to work, for example, *pull* (attract) them by the nature of the work itself.

Regarding the statistical aspects of the paper in relation to Facet Theory, the aggregative knowledge about comparing SSA and FA results has consistently pointed to the superiority of the scaling methods (i.e., SSA) over FA (e.g., Tziner, 1992; de Souza et al., 2015; Rabenu et al., 2015a). Apparently it is less appropriate to use the FA method, which is linear and unidimensional, in multidimensional data. As such, only scaling methods such as SSA (and other MDS) should be implemented. We recommend the use of cross-algorithmic validation for the scaling methods. Meaning, for example, analyzing the data using at least two different MDS algorithms (e.g., ALSCAL, SMACOF, fSSA, PROXSCAL) and comparing the results. In addition, we also suggest intra-sample validation in future studies, in which the sample is divided into at least two randomized unbiased groups and the analyses run on each one in order to replicate results group-wise and not just total-sample-wise.

We suggest formulating a refined mapping sentence and a consequent new scale for the concept of “doingism.” This mapping sentence should also include a new facet (C) titled “life-area domains” (including the elements: work, home, leisure). This would enable researchers to identify “doingistic” tendencies in general, or even potential workaholics.

### Limitations

The present research has a number of limitations. First, the Romanian sample filled out four workaholism measures in a single survey (i.e., WFBS, DUWAS, BWAS, HWI) for a total of 47 items. This might have biased the participants into a “workaholic state of mind,” but as shown in the method section the data did not suffer from CMB issues, and even the correlations between the constructs (see Table 5) were not inflated. Another possible drawback is that the research variables in this study were collected from single-source data, namely self-report questionnaires. We believe that due to the nature of the variables (the need to describe *inner* drive) subjective reports would be the most appropriate. However, we did not collect objective data that may be relevant to the instrumental items specifically. For example, the item “… arrive very early to work, and leave it very late” (WFBS, item no. 11) can be also checked objectively in the workplace hard data (e.g., time clock records).

In addition, the Romanian sample consisted of working MBA students, and half of the Israelis were managers (at various levels). Since managers have unique characteristics, and workaholism is a phenomenon typical to senior managers (Pines, 2011, p. 164), this might have biased the results.

## Ethics statement

The current study was correlational, based on a survey, and not a manipulation on subjects. At the beginning of each questionnaire, we explained the general goal of the research, ensured anonymity and discretion of the results, and also ensured the subjects know they could leave the participation at any time they choose.

## Author contributions

All authors listed have made a substantial, direct and intellectual contribution to the work, and approved it for publication.

### Conflict of interest statement

The authors declare that the research was conducted in the absence of any commercial or financial relationships that could be construed as a potential conflict of interest.
