# Supplementary material for: Organizing the Confusion Surrounding Workaholism: New Structure, Measure, and Validation
Source: Front Psychol. 2017 Oct 19;8:1803. doi: 10.3389/fpsyg.2017.01803 (PMC5654401; doi:10.3389/fpsyg.2017.01803)
Supplement: Supplementary file 1 [file AppendixA.pdf]

Appendix A

Table comparing different workaholism measures

Assessment	*Original definition*	*Subscales*	*Similarities to WFBS*	*Distinctions from WFBS*
Workaholism Battery (WorkBat; Spence & Robbins, 1992)	"…like alcoholism, workaholism is an addiction; the workaholic feels driven or compelled to work, not because of external demands or pleasure in work, but because of inner pressures that make the person distressed or guilty about not working" (Spence & Robbins, 1992, p. 161).	Work Drive (*WD*), Work Enjoyment (*WE*), Work Involvement (*WI*)	WorkBat includes the drive component as one of the core elements of workaholism (i.e.: Work Drive). WorkBat addresses (mainly) the instrumental and emotional aspects of workaholism.	In contrast to WorkBat, which includes involvement and enjoyment components, we focus solely on the drive to work as the core element of workaholism. WorkBat almost does not address the cognitive aspect of workaholism.
Work Addiction Risk Test (WART; Robinson, 1989, 1999)	"…an obsessive compulsive disorder that manifests itself through self-imposed demands, an inability to regulate work habits, and an over indulgence in work to the exclusion of most other life activities" (Robinson, 1989, p. 8)	Inability to Delegate (*ID*), Control (*C*), Compulsive Tendencies (*CT*), Self-Worth (*SW*), Impaired Communication/Self-Absorption (*IC/SA*)	One of the core-elements of defining workaholism is working compulsively (Robinson, 1989).	WART was formulated based on symptoms reported by clinicians who were treating workaholics (Andreassen, 2015, p. 82). Our measure is theoretically based. WART is 'contaminated' with too much overlapping with Type-A personality (e.g.: Andreassen, 2015; McMillan, Brady, O'Driscoll, & Marsh, 2002; Robinson, 1996; Robinson & Kelley, 1998).
Dutch Work Addiction Scale (DUWAS; Schaufeli et al., 2009)	"The tendency to work excessively hard and being obsessed with work, which manifests itself in working compulsively" (Schaufeli et al., 2009, p. 322)	Working Compulsively (*WC*), Working Excessively (*WE*)	Workaholism is considered an internal and uncontrollable drive (i.e.: Working Compulsively). The Working Excessively subscale corresponds with our Resources facet (time and effort).<br><br>DUWAS emphasizes the instrumental and emotional aspects of workaholism.	DUWAS does not broadly address cognitive items. The Working Excessively subscale is mainly instrumental in nature (lacking the other elements in our modality facet).
Bergen Work Addiction	"…being overly concerned about work, being driven by an uncontrollable work	None	In general, BWAS addresses all the aspects of workaholism	WFBS is non-judgmental as it does not addresses workaholism as a negative or a positive

Assessment	*Original definition*	*Subscales*	*Similarities to WFBS*	*Distinctions from WFBS*
Scale[1] (BWAS; Andreassen et al., 2012)	motivation, and spending energy and effort on work that it impairs private relationships, spare-time activities, and/or health" (Andreassen et al., 2014, p. 8)		(instrumental, emotional, and cognitive).	phenomenon (as hinted in BWAS items no. 3 and 7: "Worked in order to reduce feelings of guilt, anxiety, helplessness and/or depression?" and "Worked so much that it has negatively influenced your health?", respectively). WFBS is descriptive and not inferential (it asks individuals to describe their behavior, and not their reasons behind it, as hinted in item no. 3). The BWAS scale emphasizes frequency, while ours emphasizes the intensity of the work drive.

Notes: (1) Although BWAS does not directly address the drive component of workaholism, it was called an "addiction".
