# Supplementary material for: Organizing the Confusion Surrounding Workaholism: New Structure, Measure, and Validation
Source: Front Psychol. 2017 Oct 19;8:1803. doi: 10.3389/fpsyg.2017.01803 (PMC5654401; doi:10.3389/fpsyg.2017.01803)
Supplement: Supplementary file 2 [file AppendixB.pdf]

## Appendix B

*The Workaholism Facet-Based Scale (WFBS) measure*

Please read the sentences below and indicate **the extent to which an internal drive "compels" you to**:

| | | | **Strongly disagree** | **Disagree** | **Somewhat disagree** | **Somewhat agree** | **Agree** | **Strongly agree** |
|---|---|---|---|---|---|---|---|---|
| a1-b1 | **1.** | Think about the job all the time. | 1 | 2 | 3 | 4 | 5 | 6 |
| a1-b2 | **2.** | Invest vast cognitive effort regarding your job (**e.g.**: planning, examining alternatives for problem solving in the job, etc.) | 1 | 2 | 3 | 4 | 5 | 6 |
| a2-b1 | **3.** | Feel frustrated if you need to stay at work beyond the formal working hours. **(R)** | 1 | 2 | 3 | 4 | 5 | 6 |
| a2-b2 | **4.** | Feel bitter when you need to invest a lot of effort in your work. **(R)** | 1 | 2 | 3 | 4 | 5 | 6 |
| a3-b1 | **5.** | Work long hours, even beyond the role's requirements. | 1 | 2 | 3 | 4 | 5 | 6 |
| a3-b2 | **6.** | Work very diligently. | 1 | 2 | 3 | 4 | 5 | 6 |
| a1-b1 | **7.** | Think constantly about job-related issues (**e.g.**: while driving, on holidays and rest days, etc.). | 1 | 2 | 3 | 4 | 5 | 6 |
| a1-b2 | **8.** | Invest much thought regarding your work, even when you wish to disengage from it. | 1 | 2 | 3 | 4 | 5 | 6 |
| a2-b1 | **9.** | Feel anxiety if you are not occupied all day long. | 1 | 2 | 3 | 4 | 5 | 6 |
| a2-b2 | **10.** | Feel ecstatic when you need to invest much effort at work. | 1 | 2 | 3 | 4 | 5 | 6 |
| a3-b1 | **11.** | Arrive very early to work, and leave very late. | 1 | 2 | 3 | 4 | 5 | 6 |
| a3-b2 | **12.** | Work very intensely. | 1 | 2 | 3 | 4 | 5 | 6 |
| a1-b1 | **13.** | Think long about work issues. | 1 | 2 | 3 | 4 | 5 | 6 |
| a1-b2 | **14.** | Positively perceive a role in which you can infuse effort, regardless of the interest that may derive from the tasks themselves. | 1 | 2 | 3 | 4 | 5 | 6 |
| a2-b1 | **15.** | Feel disappointed when a working day ends earlier than expected. | 1 | 2 | 3 | 4 | 5 | 6 |
| a2-b2 | **16.** | Feel enthusiastic about working days, which require heavy work investment. | 1 | 2 | 3 | 4 | 5 | 6 |
| a3-b1 | **17.** | Work all the time, even on breaks (**e.g.**: lunch breaks, smoking breaks, etc.) | 1 | 2 | 3 | 4 | 5 | 6 |
| a3-b2 | **18.** | Invest a large amount of effort in executing job tasks. | 1 | 2 | 3 | 4 | 5 | 6 |

*Notes*: The left-most column shows the structuples based on the mapping sentence. An (R) symbol refers to reverse-coded items.
