# Supplementary material for: Organizing the Confusion Surrounding Workaholism: New Structure, Measure, and Validation
Source: Front Psychol. 2017 Oct 19;8:1803. doi: 10.3389/fpsyg.2017.01803 (PMC5654401; doi:10.3389/fpsyg.2017.01803)

Appendix C

CFA for SMACOF results

Fit indices for the CFA comparing Israeli (*N* = 166) and Romanian (*N* = 1,117) samples

Model	χ^2(df)	χ^2/df	SRMR	CFI	GFI	NFI	RMSEA (90% CI)
Israel	403.52 (132)[1]	3.06	.12	.76	.79	.68	.11 (.10-.12)[2]
Romania	1847.45 (131)[3]	14.10	.09	.78	.82	.77	.11 (.10-.11)[4]

Notes: (1) p = .000, (2) *p-close* = .000. (3) p = .000, (4) *p-close* = .000.

Path diagram for the CFA, Israel

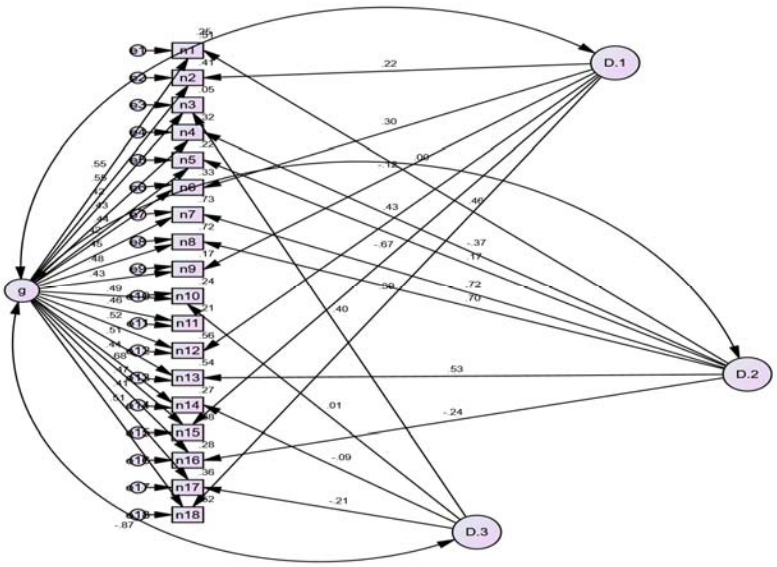

Path diagram for the CFA, Romania

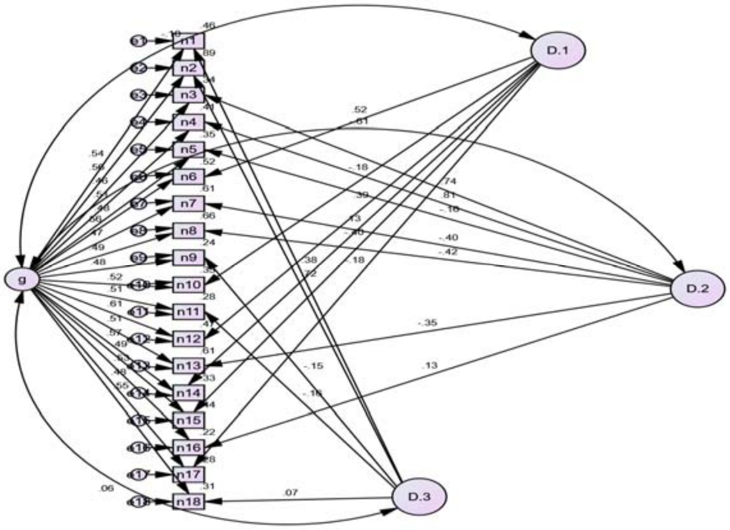

Supplement: Supplementary file 3 [file AppendixC.pdf]
